# Liraglutide ameliorates delirium-like behaviors of aged mice undergoing cardiac surgery by mitigating microglia activation via promoting mitophagy

**DOI:** 10.1007/s00213-023-06492-7

**Published:** 2023-11-16

**Authors:** Min Jia, Xin Lv, Tong Zhu, Jin-Chun Shen, Wen-xue Liu, Jian-jun Yang

**Affiliations:** 1 Department of Anesthesiology, Jinling Hospital, Affiliated Hospital of Medical School, Nanjing University, Nanjing, 210002 China; 2 Department of Anesthesiology, Pain and Perioperative Medicine, The First Affiliated Hospital of Zhengzhou University, Zhengzhou, China; 3 Department of Thoracic and Cardiovascular Surgery, Institute of Cardiothoracic Vascular Disease, Affiliated Drum Tower Hospital of Nanjing University Medical School, Nanjing University, Nanjing, China

**Keywords:** Postoperative delirium, Liraglutide, Microglia mitophagy, Synaptic plasticity, Cardiac surgery

## Abstract

**Objective:**

Postoperative delirium (POD) is a prevalent complication in cardiac surgery patients, particularly the elderly, with neuroinflammation posited as a crucial contributing factor. We investigated the prophylactic effects of liraglutide, a GLP-1 analog, on delirium-like behaviors in aged mice undergoing cardiac surgery and explored the underlying mechanisms focusing on neuroinflammation, mitochondrial dysfunction, and synaptic plasticity.

**Methods:**

Using a cardiac ischemia-reperfusion animal model to mimic cardiac surgery, we assessed delirium-like behaviors, microglial activation, NLRP3 inflammasome activation, mitophagy, synaptic engulfment, and synaptic plasticity.

**Results:**

Cardiac surgery triggered delirium-like behaviors, concomitant with heightened microglial and NLRP3 inflammasome activation and impaired mitochondrial function and synaptic plasticity. Pretreatment with liraglutide ameliorated these adverse outcomes. Mechanistically, liraglutide enhanced mitophagy, thereby inhibiting NLRP3 inflammasome activation and subsequent microglial activation. Furthermore, liraglutide counteracted surgery-induced synaptic loss and impairment of synaptic plasticity.

**Conclusion:**

Liraglutide exerts protective effects against delirium-like behaviors in aged mice post-cardiac surgery, potentially through bolstering microglia mitophagy, curtailing neuroinflammation, and preserving synaptic integrity. This highlights the potential of liraglutide as a promising perioperative strategy for delirium prevention in cardiac surgery patients.

## Introduction

Delirium, an acute brain illness, manifests through changes in consciousness, attention, cognition, and perception (Djaiani et al. 2016). Among patients undergoing cardiac surgery, the occurrence of postoperative delirium (POD) ranges from 20 to 50%, with the elderly being particularly vulnerable (Inouye et al. 2014; Katznelson et al. 2009; Sugawara et al. 2022; van Norden et al. 2021). This distressing condition not only impacts patients but also takes a toll on their families, and is associated with heightened morbidity, mortality, extended hospital stays, functional and long-term cognitive decline, and escalated healthcare costs (Ely et al. 2001, 2004; Marcantonio 2017). Despite a clear understanding of the risk factors and consequences of POD, effective perioperative strategies for delirium prevention remain elusive (Mattison 2020). The escalating global aging trend has led to a surge in elderly cardiac surgery patients (Carrascal 2007), underscoring the urgent need to delve into the molecular mechanisms underlying postoperative delirium and identify the intervention targets that can aid in its prevention and treatment.

During the past few decades, multiple factors have been identified as potential contributors to the onset of delirium, including neuroinflammation, network dysconnectivity, neurotransmitter imbalances, excess cortisol, and so on (Gaudreau 2012; Maldonado 2013). Among these factors, neuroinflammation stands out as particularly intriguing. This is due to the existing evidence linking neuroinflammation with dementia (Smirnov and Galasko 2022), a condition characterized by chronic cognitive impairment. Notably, dementia has also been recognized as a risk factor for the development of delirium (Fong et al. 2015; Fong and Inouye 2022). Consequently, the concept that neuroinflammation might play a role in the onset of delirium, which represents an acute cognitive impairment, is entirely plausible.

Microglia, belonging to the myeloid lineage of innate immune cells and located within the central nervous system (CNS), assume a pivotal role in the context of neuroinflammation (Leng and Edison 2021). A wealth of studies have highlighted the activation of microglia following systemic inflammation triggered by surgical procedures, subsequently leading to processes involving synaptic elimination and cognitive dysfunction (Chen et al. 2019; Leng and Edison 2021). The NOD-like receptor protein 3 (NLRP3) inflammasome, recognized as a key player among innate immune sensors, acts as the primary platform for caspase-1 activation and the maturation of the proinflammatory cytokine IL-1 (Lamkanfi and Dixit 2014). There is substantial evidence demonstrating that NLRP3 is prominently expressed in microglia, and its excessive formation is implicated in both the activation of microglia and the pathogenesis of numerous brain disorders (de Rivero Vaccari et al. 2014; Heneka et al. 2018). Furthermore, anesthesia and surgical procedures can lead to damage in mitochondria (Netto et al. 2018), which is then specifically addressed through the PINK1/Parkin mediated mitophagy (Heo et al. 2018). However, any impairment or insufficiency in mitophagy can result in the accumulation of dysfunctional mitochondria and subsequently trigger the activation of the NLRP3 inflammasome (Mishra et al. 2021).

Glucagon-like peptide-1 (GLP-1) serves as an innate incretin hormone, secreted by L cells in the small intestine upon food intake (Campbell and Drucker 2013). It plays a multifaceted role in regulating cellular metabolism by binding to GLP-1 receptors (GLP-1R). Liraglutide, a long-acting analog of GLP-1, is currently employed to manage type 2 diabetes mellitus (T2DM) (Imprialos et al. 2017). Beyond its primary function of glycemic control, emerging evidence highlights liraglutide’s capacity for exerting neurotrophic and neuroprotective effects in the domains of neurodegeneration and neurogenesis (Hölscher 2014; Mansur et al. 2017; Zhang et al. 2019). Furthermore, prior investigations have shown liraglutide’s ability to amplify mitophagy and attenuate NLRP3 inflammasome activation in hepatocytes and cardiomyocytes (Chen et al. 2018; Hölscher 2014). However, whether liraglutide can decrease the activation of microglia by augmenting mitophagy and inhibitiing NLRP3 inflammasome, and subsequently alleviate delirium-like behaviors in elderly mice undergoing cardiac surgery remains to be elucidated.

## Methods and materials

### Animals and myocardial ischemia-reperfusion (IR) surgery

All experiments adhered to the guidelines set by the Animal Care and Use Committee of the National Institutes of Health (MD, USA). The study protocol was approved by the Ethics Committee of Nanjing Drum Tower Hospital, Affiliated Hospital of Medical School, Nanjing University (Nanjing, China). Male C57BL/6 mice aged 18–20 months were sourced from Aniphe Biolaboratory Inc. (Nanjing, China). Throughout the study, mice were group-housed and sustained in a controlled environment featuring a 12:12 light/dark cycle, temperature of 22 ± 1 °C, and 50 ± 10% humidity, with continuous access to food and water.

The myocardial IR procedure was adapted from the established method with slight changes (Curaj et al. 2015). In brief, mice were anesthetized using isoflurane and ventilated with a specialized animal ventilator (SAR-830/AP, CWE, Inc, PA, USA). A lateral incision was made between the fourth and fifth left ribs to expose the left ventricle. The left anterior descending artery (LAD) was then ligated with a 7 − 0 silk suture and released after 45 min. Visual confirmation of ischemia and reperfusion was achieved by observing heart muscle coloration and contractile variations through a stereomicroscope. Subsequently, the chest and skin were stitched separately.

### Behavioral tests

24 h post-IR surgery, the mice underwent sequential behavioral assessments: open field test (OFT), elevated plus maze test (EPM), Y-maze test (YMT), and novel object recognition test (NORT). We maintained a gap of over an hour between each test to ensure reliable results. Using the automated video tracking system (XR-Xmaze, Shanghai Xinruan Information Technology Co., LTD, Shanghai, China), we tracked and analyzed each mouse’s behavior. To avoid interference from olfactory cues, chambers received thorough cleaning with 30% ethanol and were subsequently dried using paper towels ahead of each test.

### Open field test (OFT)

For the OFT, mice were introduced to the center of an open field chamber and left to freely move for 5 min. We measured their total distance traveled to gauge their overall activity.

### Elevated plus maze test (EPM)

The EPM served as a means to detect anxiety-related behaviors. Mice were delicately positioned at the maze’s intersection, oriented towards an open arm opposite the experimenter’s location. With the help of an overhead video camera, their activities were observed and logged for 5 min. We documented the duration spent in both the closed (CA) and open arms (OA), subsequently calculating the percentage of time they spent in the OA.

### Y maze test (YMT)

YMT evaluated the mice’s short-term spatial memory. This three-armed maze, with each arm placed at a 120° angle relative to the others, allowed us to observe the mouse’s movement. When a mouse’s four limbs were inside an arm, it was considered a single entry. We documented the total arm entries and successful alternations, then computed the alternation score based on the formula: (number of successful alternations / (total arm entries − 2)) × 100.

### Novel objective recognition test (NORT)

The NORT aimed to discern the recognition memory capacities of the mice. Before the actual test, mice were familiarized with the experimental setting 24 h in advance. The process was divided into two sessions: training and testing. During training, mice interacted with two identical objects in an open chamber for 10 min. The testing phase, conducted 24 h post-training, reintroduced the mice to the chamber, now containing one familiar and one new object, positioned as in the training. Using our video system, we monitored the exploration durations and derived the discrimination index by comparing the time spent with the novel object to the cumulative time spent with both objects.

### Immunofluorescence staining (IF)

The mice were anesthetized by isoflurane and subsequently perfused with phosphate buffer saline (PBS) and phosphate-buffered 4% paraformaldehyde (PFA) via a transcardial method. Brains were immersed in 4% PFA for further post-fixation overnight, dehydrated with 30% sucrose, embedded in OCT, and stored at -80 °C. A frozen section machine (CM1950, Leica) cut the hippocampal sections into 30 μm slices coronally. Brain slices were washed with PBS for 3 times lasting 30 min followed by being blocked in 10% normal goat or donkey serum in TBST with 0.3% Triton X-100, and then primary antibodies in blocking buffer were applied overnight at 4 °C. The primary antibodies were used as follow: IBA1 (1:500, Rabbit, ab178846, Abcam), IBA1 (1:500, Goat, ab289874, Abcam), CD68 (1:500, Rat, BioLegend, 137,001), NLRP3 (1:500, Rabbit, ab270449, Abcam), PINK1 (1:200, Mouse, sc518052, Santa Cruz), Parkin (1:200, Mouse, sc32282, Santa Cruz), PSD95 (1:500, Mouse, ab13552, Abcam). The next day, slices were washed with PBS and incubated with the species-appropriate secondary antibodies. The secondary antibodies were all used as follows: Goat Anti-Rabbit Alexa Fluor 488 (1:1000, Ab150077, Abcam), Donkey Anti-Goat Alexa Fluor 647 (1:1000, Ab150135, Abcam), Goat Anti-Mouse Alexa Fluor 594 (1:1000, Abcam, Ab150113), Goat Anti-Rat Alexa Fluor 594 (1:1000, Abcam, Ab150160). Then, slices were washed with PBS and mounted with DAPI (1:1000, D8417, sigma) into PBS with 50% sterile glycerol. The images of the hippocampal CA1 region were captured by Olympus confocal microscope FV3000 with z-stack using a 40x oil objective. The 3D-reconstructed using the surface rendering function in Imaris 10.0.0. The CD68 or PSD95 inside IBA1 were filtered using the shortest distance to surfaces function, and the volume fraction was then quantified. The fluorescence intensity of NLRP3, PINK1, and Parkin in microglia was measured by Fiji software using the threshold method combined region of interest manager.

### Western blot (WB)

The hippocampus was rapidly harvested after the mice were anesthetized by isoflurane. All procedures were performed on ice. Briefly, the hippocampus was homogenized in RIPA lysis buffer (50 mM Tris-HCl, 1% Triton X-100, 150 mM NaCl, 0.1% SDS) plus 0.5% Na3VO4, 10 mM NaF, 1 mM PMSF and protease inhibitor cocktail tablets, shake gently at 4 °C for 30 min, centrifuge at 10,000 g for 30 min at 4 °C, and carefully collect the supernatant. Protein concentration was determined by bicinchoninic acid (BCA) protein assay kit (23,225, Thermo Fisher Scientific, Waltham, MA, USA), and then, the samples were adjusted to an equal protein concentration with protein lysis buffer and 5× sample loading buffer. Samples were then boiled at 95 °C for 5 min and stored at -20 °C before the immunoblot analysis. Equal amounts of proteins in each group were added and separated by SDS/PAGE and transferred to the PVDF membrane. After that, the membrane was blocked by 5% non-fat milk in 1× Tris-buffered saline containing 0.1% Tween-20 (TBST) for 1 h at room temperature. The incubation of the following primary antibodies was carried out overnight at 4 °C: NLRP3 (1:500, Rabbit, ab270449, Abcam), Caspase-1 (1:500, Rabbit, 24,232, CST), IL-1β (1:500, Mouse, 12,153, CST). PINK1 (1:500, Mouse, sc518052, Santa Cruz), Parkin (1:500, Mouse, sc32282, Santa Cruz), And then, the membrane were washed with TBST for 3 × 5 min, then incubated with species-appropriate HRP-conjugated secondary antibodies in 5% non-fat for 1 h at room temperature. After incubation, the membrane was washed with TBST for 3 × 5 min, and developed with enhanced chemiluminescence reagent. The protein expression level was quantified using Fiji software (1.54f, NIH, Bethesda, MD, USA).

### Separation of hippocampal mitochondria, detection of mitochondrial membrane potential, and ATP assay

Tissue mitochondrial extraction kits (C3606, Beyotime, Shanghai, China) were applied for hippocampal mitochondrial separation according to the manufacturer’s instructions and used for further detection of mitochondrial membrane potential, and ATP assay. Mitochondrial membrane potential was evaluated by enhanced mitochondrial membrane potential assay kit with JC-1 staining kit (C2003S, Beyotime, Shanghai, China); the ATP assay was performed by the ATP Assay Kit (S0026, Beyotime, Shanghai, China) was used to measure the ATP levels of the hippocampus.

### Golgi staining

The mice were anesthetized by isoflurane and the brains were rapidly harvested, washed with Milli-Q water, and immersed in buffers from FD Rapid GolgiStainTM Kit (FD Neurotechnologies, Inc., Columbia, Maryland) according to our previous report. Serial brain slices (110 μm) were cut by using a Vibratome (VT3000, Leica) and stained according to the manual of FD Rapid GolgiStainTM Kit. Bright-field microscopy (Olympus) with a 10 × objective at a magnification of 20× and 40× were taken the pyramidal neurons and dendritic morphology in the CA1 region by an observer blinded to the experiment, respectively.

For the hippocampal CA1 regions, apical dendrites on representative pyramidal neurons were sampled for the analysis. The tracings of neurons and quantification of dendritic total length, the number of branching points, and intersections at concentric circles at 50 μm intervals from the soma center were performed using Neuron J (version 1.4.3) and Sholl analysis (version v3.4.4, NIH, Bethesda, MD) of image J software plugins, respectively. The number of spines was counted by using FIJI software.

### Electrophysiology recording

Mice were anesthetized using isoflurane and subsequently perfused transcardially with cold cutting solution, bubbled with 95% O2 and 5% CO2. This solution comprised (in mM): 93 NMDG, 2.5 KCl, 1.2 NaH2PO4, 30 NaHCO3, 20 HEPES, 25 glucose, 2 thiourea, 5 Na-ascorbate, 3 Na-pyruvate, 0.5 CaCl2, and 10 MgSO4. Following perfusion, the brain was swiftly extracted, and transverse hippocampal slices of 400 μm thickness were prepared. These slices were incubated in the aforementioned solution, enriched with 12 mM NAC for 12 min at 30℃. Afterward, they were transferred to ACSF, bubbled with 95% O2 and 5% CO2, for an hour—half of this duration at 30℃ and the remainder at room temperature. The ACSF composition was (in mM): 92 NaCl, 2.5 KCl, 30 NaHCO3, 1.2 NaH2PO4, 25 glucose, 20 HEPES, 5 Na-ascorbate, 3 Na-pyruvate, 2 Thiourea, 2 CaCl2, and 2 MgCl2.

Recordings were conducted in a chamber with ACSF at 30 ± 1℃. Evoked fEPSPs were captured using a micropipette positioned within the hippocampal CA1 region’s stratum radiatum. A bipolar electrode stimulated the Schaffer-collaterals in the CA1. After setting a baseline for fEPSPs, the LTP protocol was initiated with 4 high-frequency stimulation trains. Post LTP induction, slope alterations were normalized to the average fEPSP slope observed during baseline, which was set at 100%. The LTP magnitude was determined by averaging responses recorded between 50 and 60 min post-HFS.

### Statistical analysis

All analyses were conducted using GraphPad Prism 9.0 (version 9.4.0). Data sets underwent evaluation with one-way ANOVA or two-way ANOVA followed by Tukey’s post hoc test. Results are displayed as mean ± SEM. A P-value < 0.05 indicated statistical significance.

## Results

### Liraglutide mitigates delirium-like behaviors in aged mice post cardiac surgery

**Fig. 1:**
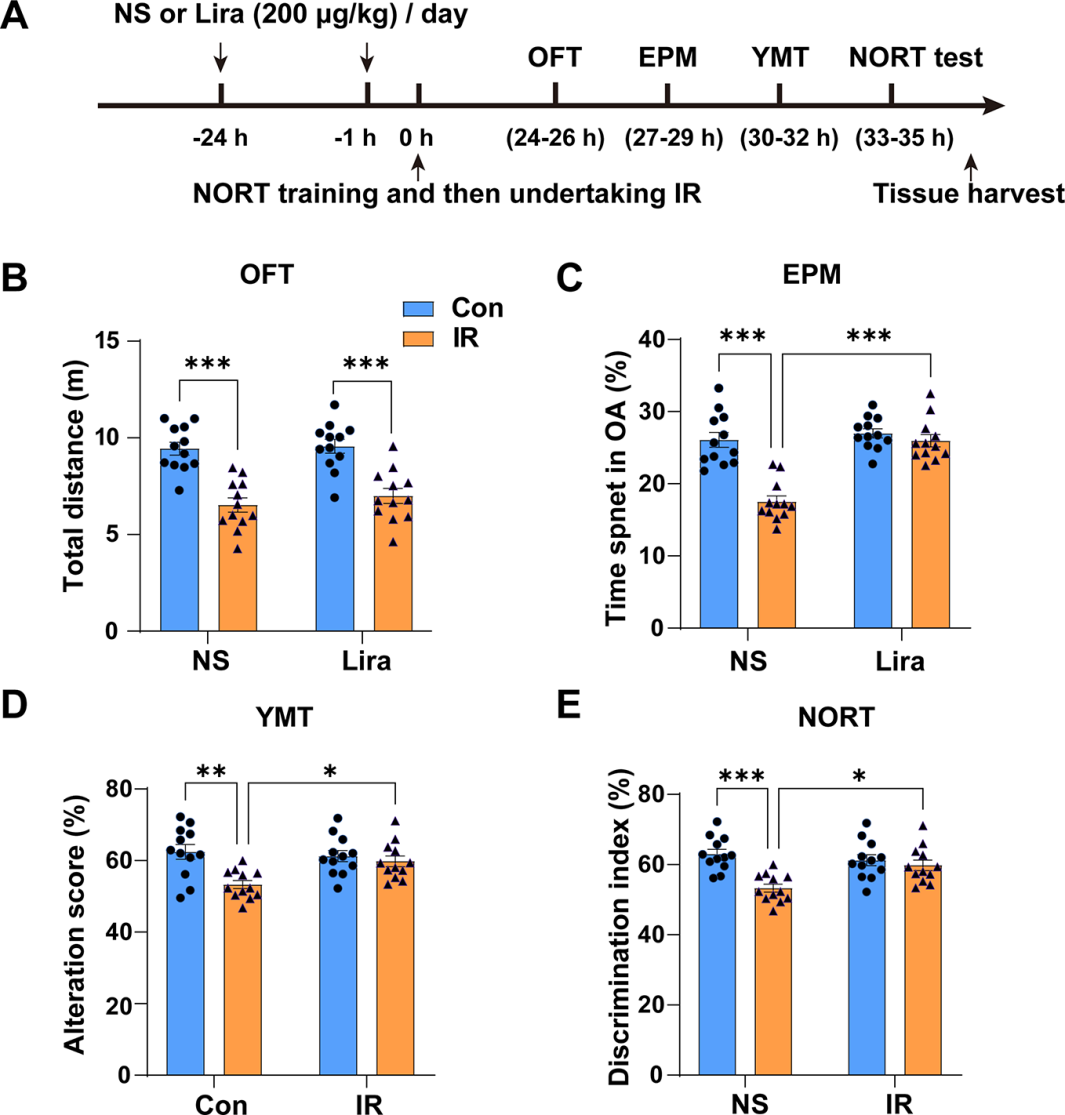
**Liraglutide mitigates delirium-like behaviors in aged mice post cardiac surgery**. **(A)** Experiment procedure for the study. **B** Total distance in the Open Field Test (OFT) assessing motion ability across groups. Two-way ANOVA: Interaction, F (1,44) = 0.2303, *P =* 0.6337; Row Factor, F (1,44) = 0.6628, *P =* 0.42; Column Factor, F (1,44) = 56.98, *P* < 0.0001. **C** Percentage of time spent in the open arm (OA) in the Elevated Plus Maze (EPM) measuring anxiety-like behaviors across groups. Two-way ANOVA: Interaction, F (1,44) = 19.96, *P* < 0.0001; Row Factor, F (1,44) = 30.69, *P* < 0.0001; Column Factor, F (1,44) = 32.32, *P* < 0.0001.** D, E **Alteration score in the Y Maze Test (YMT) (D) and discrimination index in the Novel Objective Recognition Test (NORT) (E) evaluating memory ability across groups. Two-way ANOVA: For **D**, Interaction, F (1,44) = 5.821, *P* = 0.0201; Row Factor, F (1,44) = 2.758, *P* = 0.1039; Column Factor, F (1,44) = 10.92, *P* = 0.0019; For **E**, Interaction, F (1,44) = 8.749, *P* = 0.005; Row Factor, F (1,44) = 2.839, *P* = 0.0991; Column Factor, F (1,44) = 15.80, *P* = 0.0003. Data are expressed as mean ± SEM (n = 12). *P < 0.05, **P < 0.01, ***P < 0.001

Our study’s procedures are depicted in Fig. 1A. The drug dosage and treatment cycle were determined based on previous studies (An et al. 2023; Han et al. 2019; Hulst et al. 2020a, b) and our preliminary study. Building on prior studies, we utilized a cardiac ischemia-reperfusion (IR) animal model to emulate cardiac surgery. Delirium-like behavior was assessed 24 h post-surgery using a series of behavioral tests including OFT, EPM, YMZ, and NORT. In comparison to the Con + NS group, the IR + NS group exhibited significant declines in total distance (Fig. 1B), time spent in OA (Fig. 1C), alteration scores (Fig. 1D), and NORT discrimination index (Fig. 1E). Liraglutide treatment restored these metrics, excluding total distance. These findings suggest liraglutide’s potential to alleviate delirium-like behaviors in aged mice post-cardiac surgery.

### Liraglutide suppresses microglial activation triggered by cardiac surgery

**Fig. 2:**
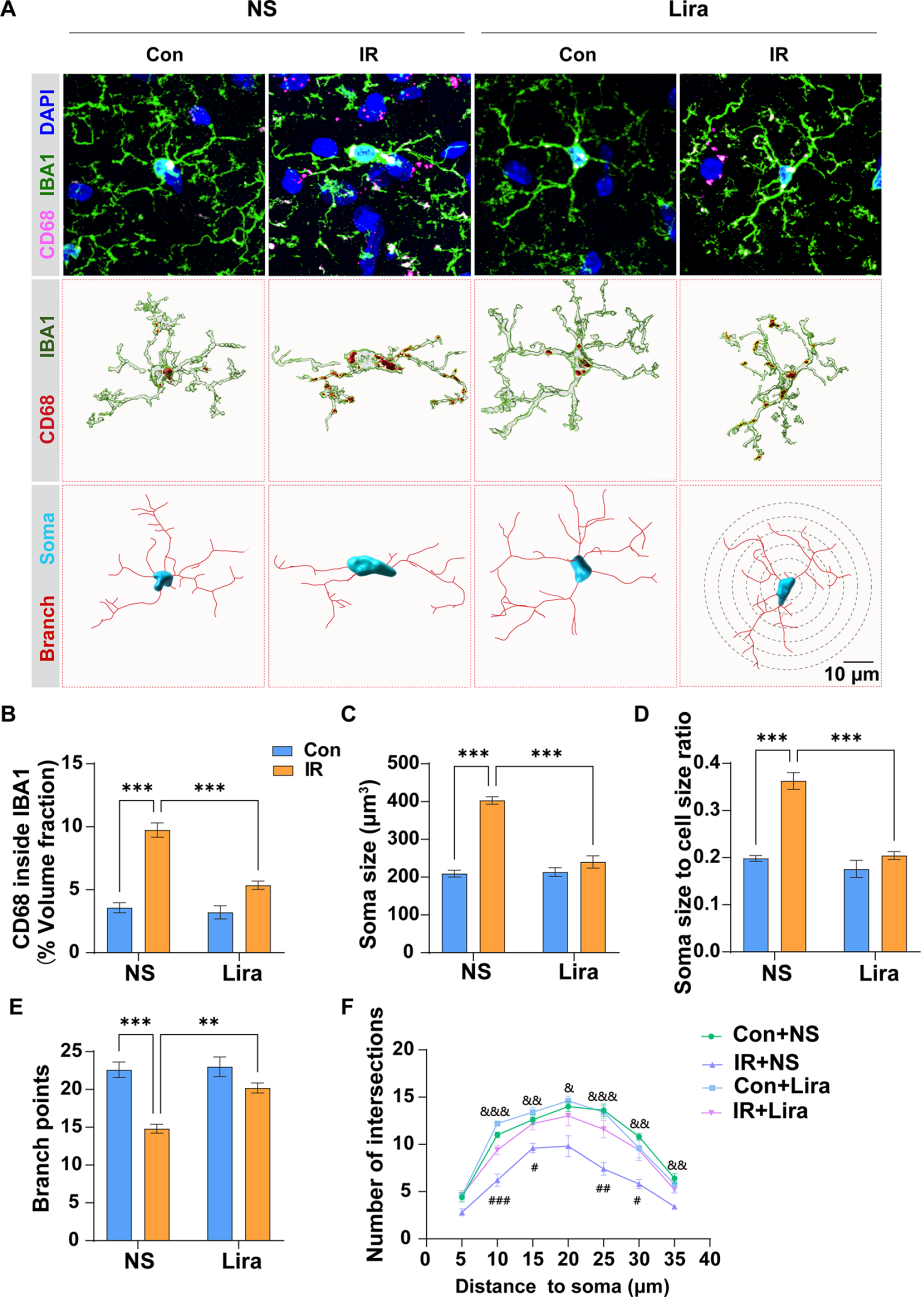
**Liraglutide suppresses microglial activation triggered by cardiac surgery**. **(A)** Representative immunofluorescence (IF) images of IBA1-positive microglia and their 3D reconstruction using Imaris. **B–F** Analysis and quantification of microglial activation and morphological parameters, including volume fraction of CD68 to IBA1 (B), soma volume (C), soma size to cell size ratio (D), branch points (E), and the number of intersections (F). Two-way ANOVA: For **B**, Interaction, F (1,16) = 18.65, *P* = 0.0005, Row Factor, F (1,16) = 25.97, *P* = 0.0001; Column Factor, F (1,16) = 80.11, *P* < 0.0001; For **C**, Interaction, F (1,16) = 14.93, *P* = 0.0014, Row Factor, F (1,16) = 23.12, *P* = 0.0002; Column Factor, F (1,16) = 51.02, *P* < 0.0001; For **D**, Interaction, F (1,16) = 7.062, *P* = 0.0172, Row Factor, F (1,16) = 9.503, *P* = 0.0071; Column Factor, F (1,16) = 31.74, *P* < 0.0001; For **E**, Interaction, F (1,16) = 48.58, *P* < 0.0001, Row Factor, F (1,16) = 43.82, *P* < 0.0001; Column Factor, F (1,16) = 84.78, *P* < 0.0001; One-way ANOVA for **F**. Data are expressed as mean ± SEM (n = 5). *P < 0.05, **P < 0.01, ***P < 0.001; Con + NS compared with IR + NS, ^&^*P* < 0.05, ^&&^*P* < 0.01, ^&&&^*P* < 0.001; IR + NS compared with IR + Lira, ^#^*P* < 0.05, ^##^*P* < 0.01, ^###^*P* < 0.001

We employed IF staining and 3D construction via Imaris to investigate microglial activation. Significant reductions in the CD68 to IBA1 volume fraction **(**Fig. 2A), soma volume **(**Fig. 2B**)**, some size to cell size ratio (Fig. 2 C), branch points, and number of intersections in the sholl analysis of microglia were observed in the IR + NS group relative to the Con + NS group. Liraglutide administration effectively reversed these changes, highlighting its role in curbing microglial activation post-cardiac surgery **(**Fig. 2A F**)**.

### Liraglutide inhibits NLRP3 inflammasome activation in microglia

**Fig. 3:**
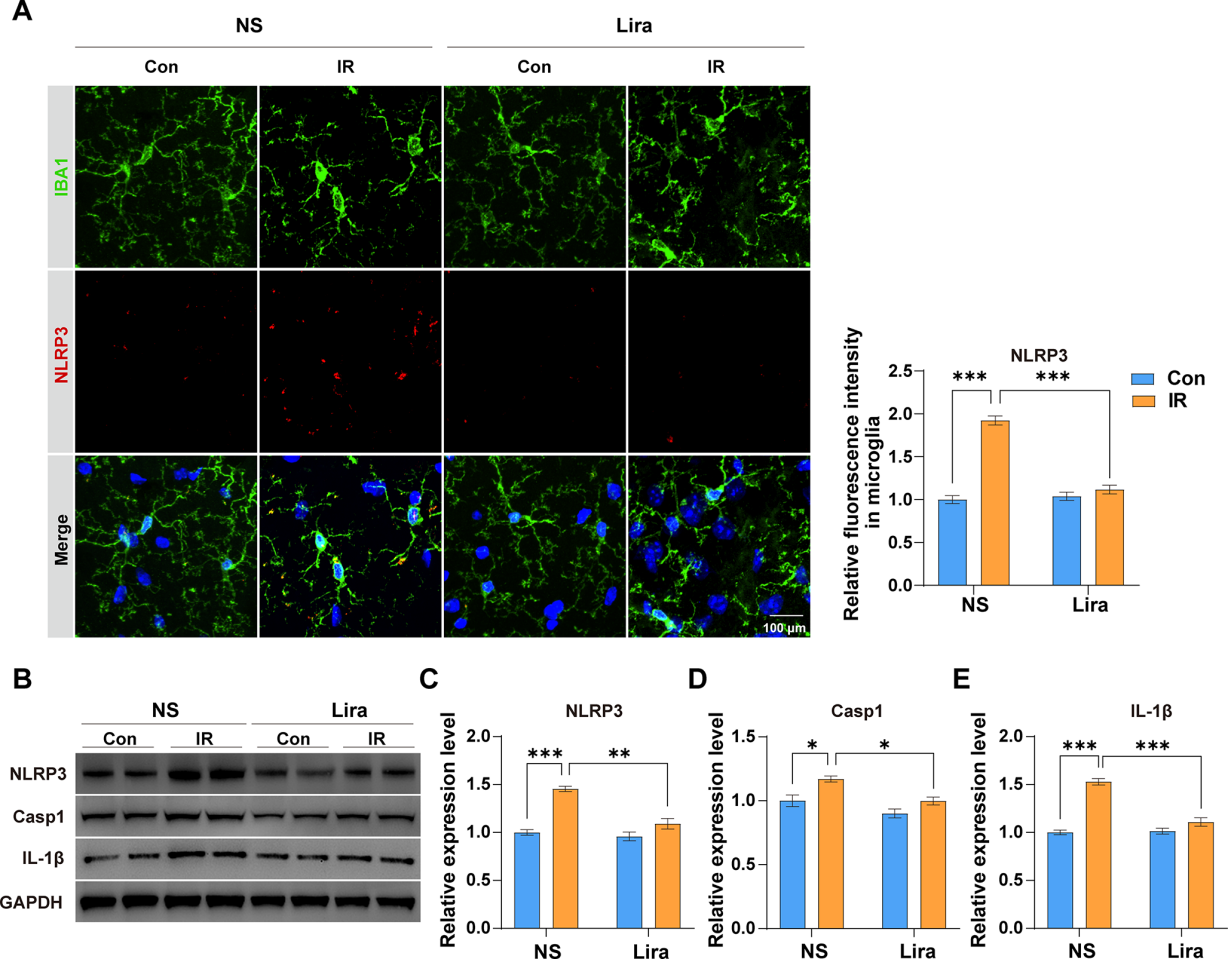
**Liraglutide inhibits NLRP3 inflammasome activation in microglia**. **(A)** Representative IF images of IBA1 and NLRP3 double staining, and the relative fluorescence intensity of NLRP3 in microglia. Two-way ANOVA: Interaction, F (1,16) = 71.84, *P* < 0.0001, Row Factor, F (1,16) = 59.4, *P* < 0.0001; Column Factor, F (1,16) = 101.1, *P* < 0.0001. **B** Representative western blot (WB) images for NLRP3, Casp1, IL-1β, and GAPDH. ** C–E** Analysis and quantification of the proteins expression level of NLRP3 (C), Casp1 (D) and IL-1β (E). Two-way ANOVA: For **C**, Interaction, F (1,8) = 15.25, *P* = 0.0045, Row Factor, F (1, 8) = 24.22, *P* = 0.0012; Column Factor, F (1, 8) = 51.06, *P* < 0.0001; For **D**, Interaction, F (1,8) = 1.128, *P* = 0.3192, Row Factor, F (1, 8) = 15.46, *P* = 0.0043; Column Factor, F (1, 8) = 15.13, *P* = 0.0046; For **E**, Interaction, F (1,8) = 39.9, *P* = 0.0002, Row Factor, F (1, 8) = 35.07, *P* = 0.0004; Column Factor, F (1, 8) = 83.24, *P* < 0.0001. Data are expressed as mean ± SEM (n = 5 for IF, n = 3 for WB). *P < 0.05, **P < 0.01, ***P < 0.001

Given the NLRP3 inflammasome’s critical role in microglial activation and numerous neurodegenerative diseases, we utilized IF staining and western blotting to gauge its activation. Relative to the Con + NS group, the IR + NS group exhibited heightened NLRP3 fluorescence intensity in microglia (Fig. 3A) and increased protein expression of NLRP3, Casp1, and IL-1β in the hippocampus **(**Fig. 3B∓E). Liraglutide significantly mitigated these changes, underscoring its potential prophylactic benefits.

### Liraglutide boosts Mitophagy in Microglia and enhances mitochondrial function

**Fig. 4:**
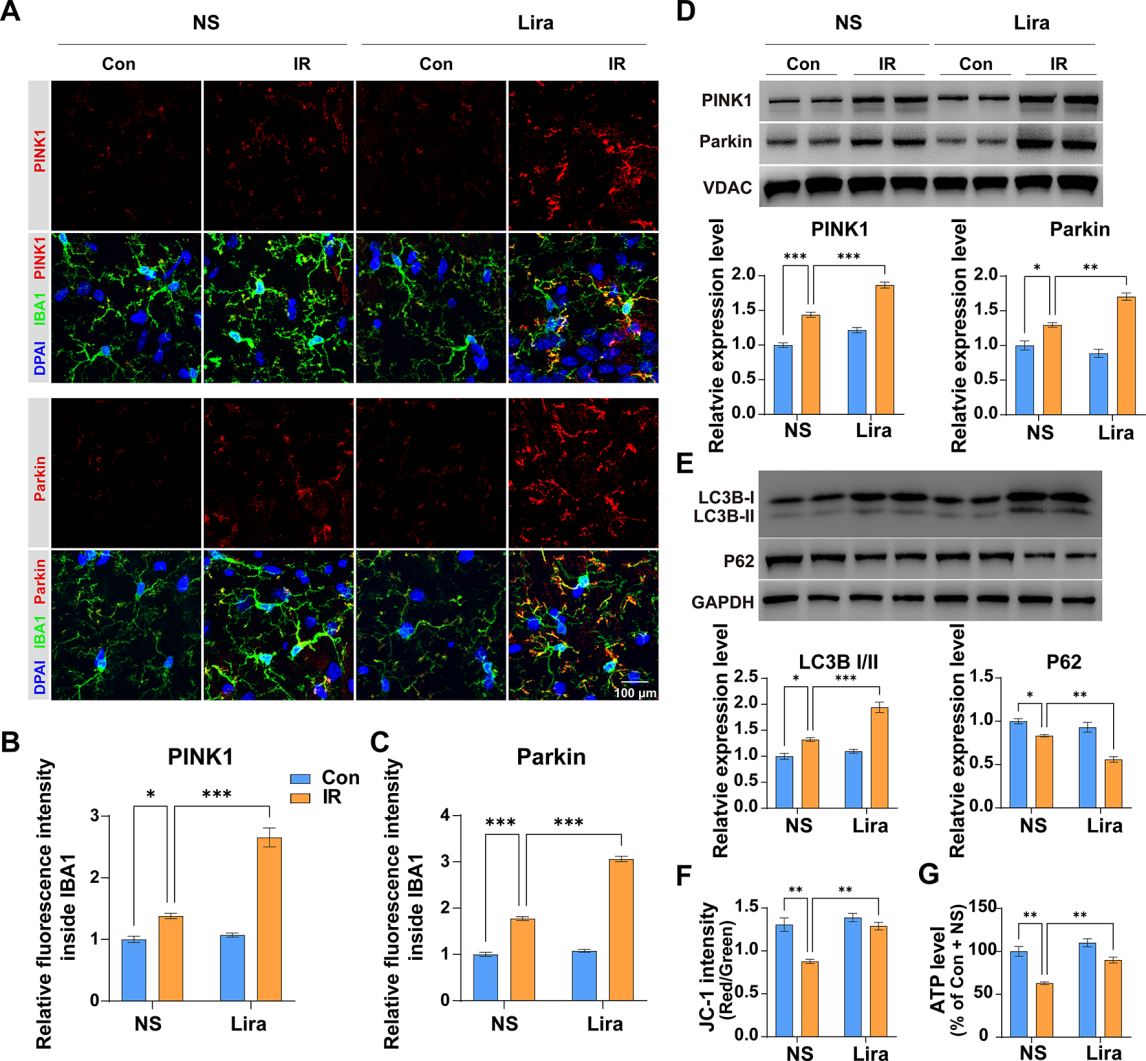
**Liraglutide boosts mitophagy in microglia and enhances mitochondrial function**. **(A)** Representative immunofluorescence images for PINK1 and IBA1, Parkin and IBA1 double staining. **B–C** Analysis and quantification of PINK1 (B) and Parkin (C) fluorescence intensity in microglia. Two-way ANOVA: For **B**, Interaction, F (1,16) = 49.46, *P* < 0.0001, Row Factor, F (1, 16) = 61.92, *P* < 0.0001; Column Factor, F (1, 16) = 131.8, *P* < 0.0001; For **C**, Interaction, F (1,16) = 170.9, *P* < 0.0001, Row Factor, F (1, 16) = 218.3, *P* < 0.0001; Column Factor, F (1, 16) = 890, *P* < 0.0001. **D** Analysis and quantification of the proteins expression level of PINK1 and Parkin in separated mitochondria. Two-way ANOVA: For PINK1, Interaction, F (1, 8) = 7.771, *P* = 0.0236, Row Factor, F (1, 8) = 70.74, *P* < 0.0001; Column Factor, F (1, 8) = 200.5, *P* < 0.0001;: For Parkin, Interaction, F (1, 8) = 23.11, *P* = 0.0013, Row Factor, F (1, 8) = 7.348, *P* = 0.0266; Column Factor, F (1, 8) = 105, *P* < 0.0001. **E** Analysis and quantification of the proteins expression level of LC3B II/I and P62 in the hippocampus. Two-way ANOVA: For LC3B II/I, Interaction, F (1, 8) = 17.33, *P* = 0.0032, Row Factor, F (1, 8) = 32.5, *P* = 0.0005; Column Factor, F (1, 8) = 85.53, *P* < 0.0001; For **P62**, Interaction, F (1, 8) = 7.916, *P* = 0.0227, Row Factor, F (1, 8) = 22.09, *P* = 0.0015; Column Factor, F (1, 8) = 54.47, *P* < 0.0001. **F** Assessment of mitochondrial membrane potentiation using JC-1 staining. Two-way ANOVA, Interaction, F (1, 8) = 9.864, *P* = 0.0138, Row Factor, F (1, 8) = 22.25, *P* = 0.0015; Column Factor, F (1, 8) = 25.66, *P* < 0.0001. **(G)** Assessment ATP level of hippocampus. Two-way ANOVA, Interaction, F (1, 8) = 4.219, *P* = 0.074, Row Factor, F (1, 8) = 19.91, *P* = 0.0021; Column Factor, F (1, 8) = 47.68, *P* = 0.0001. Data are expressed as mean ± SEM (n = 5 for IF, n = 3 for WB, JC-1 staining and ATP assay). *P < 0.05, **P < 0.01, ***P < 0.001

Mitophagy’s ability to purge damaged mitochondria, reducing ROS accumulation and NLRP3 inflammasome activation, has been well-documented. Our findings reveal increased fluorescence intensity of mitophagy mediators, PINK1 and Parkin, in microglia in the IR + NS group (Fig. 4A∓C), further amplified in the IR + Lira group. Protein levels of PINK1 and Parkin in isolated mitochondria, and LC3B II/I and P62 in the hippocampus also increased post-surgery **(**Fig. 4D and E**)**, with liraglutide further enhancing these metrics. Additionally, liraglutide counteracted the surgical-induced mitochondrial dysfunctions, as evidenced by membrane potential and ATP synthesis restoration **(**Fig. 4F and G**)**. These data indicate that liraglutide can preserve mitochondrial function by regulating mitophagy.

### Liraglutide mitigates microglial engulfment of synapses

**Fig. 5:**
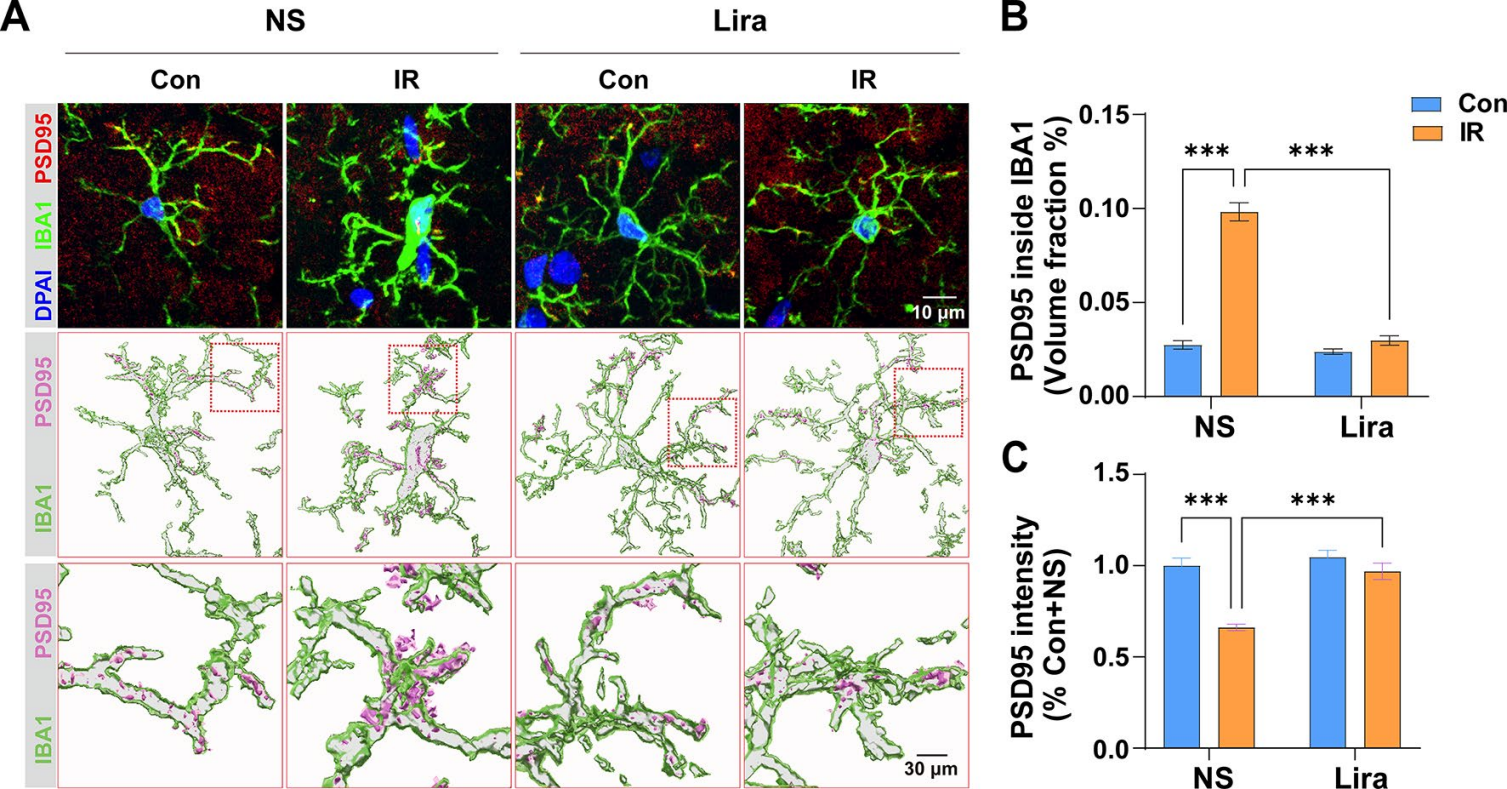
**Liraglutide mitigates microglial engulfment of synapses**. **(A)** Representative immunofluorescence images for IBA1 and PSD95 double staining and the 3D reconstructions using Imaris. **(B)** Quantification of volume fraction of PSD95 to IBA1. Two-way ANOVA, Interaction, F (1, 16) = 115.1, *P* < 0.0001, Row Factor, F (1, 16) = 141.8, *P* < 0.0001; Column Factor, F (1, 16) = 161.5, *P* < 0.0001. **(C)** Quantification of PSD95 fluorescence intensity. Two-way ANOVA, Interaction, F (1, 16) = 12.47, *P* = 0.0028, Row Factor, F (1, 16) = 23.02, *P* = 0.0002; Column Factor, F (1, 16) = 31.67, *P* < 0.0001. Data are expressed as mean ± SEM (n = 5). *P < 0.05, **P < 0.001

Activated microglia engulfment of synapses plays a pivotal role in cognitive dysfunctions. Using IF staining and 3D construction via Imaris, our data reveals a surge in synapse engulfment post-surgery, evidenced by increased engulfed PSD95 within IBA1 **(**Fig. 5A and B), and a concomitant decline in PSD95 fluorescence intensity (Fig. 5A C). Liraglutide effectively countered these changes. These results show that liraglutide can reduce synaptic elimination caused by the engulfment of activated microglia following cardiac surgery.

### Liraglutide reverses cardiac surgery-induced synaptic plasticity impairments

**Fig. 6:**
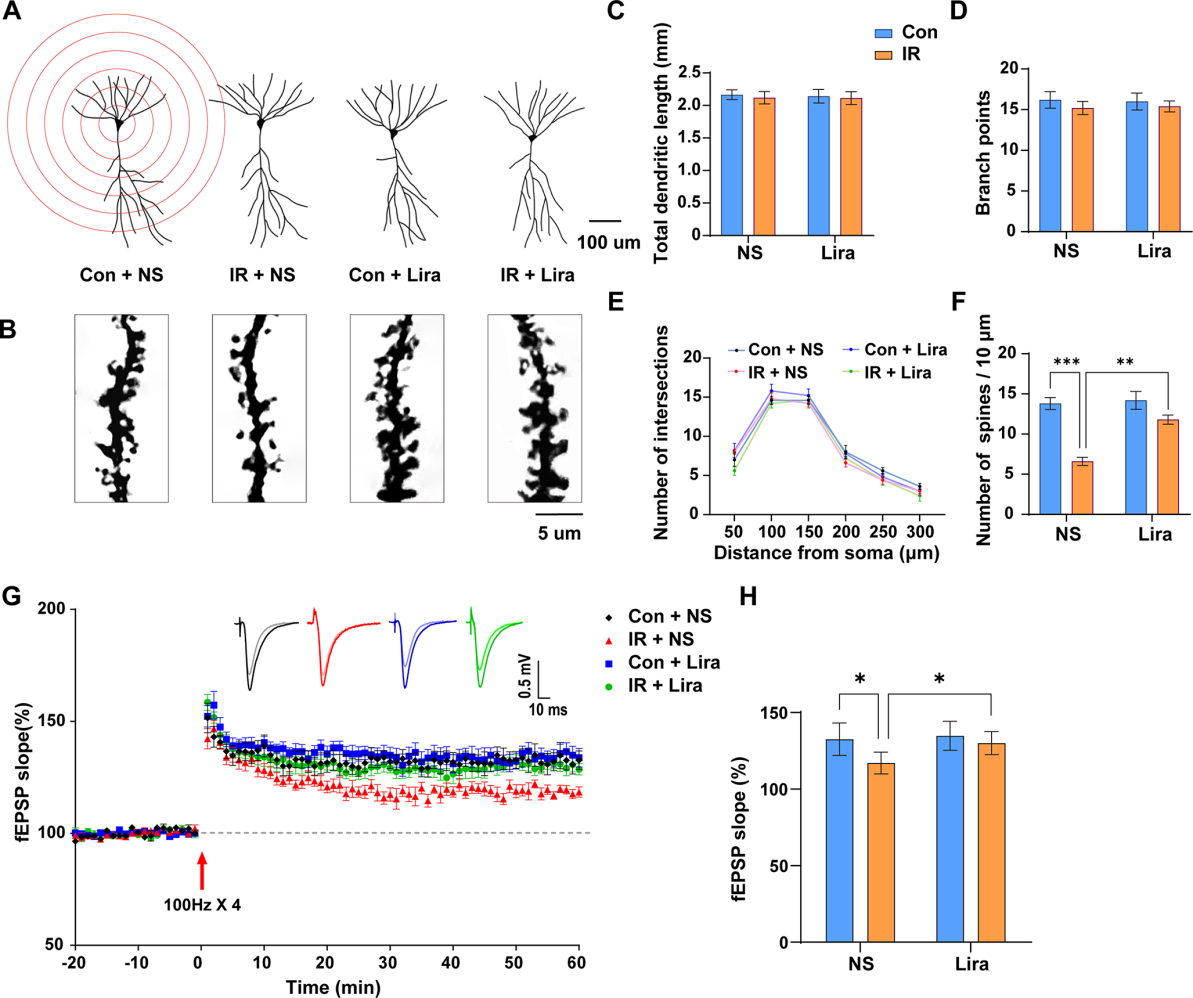
**Liraglutide reverses cardiac surgery-induced synaptic plasticity impairments**. **(A)** Representative images of hippocampal neuronal tracings and sholl analysis. **(B)** Representative images of apical dendritic spines. **(C–F)** Analysis and quantification of dendritic length (C), number of branch points (D), intersections (E) and spines (F). Two-way ANOVA: For **F**, Interaction, F (1, 16) = 9.681, *P* = 0.0067, Row Factor, F (1, 16) = 13.18, *P* = 0.0023; Column Factor, F (1, 16) = 38.72, *P* < 0.0001. **(G)** Long-term potentiation (LTP) induced by 4 trains of 100 Hz tetanic stimulus across groups. **(H)** Quantification of LTP level across groups. Two-way ANOVA, Interaction, F (1, 23) = 2.587, *P* = 0.1214, Row Factor, F (1, 23) = 5.169, *P* = 0.0326; Column Factor, F (1, 23) = 9.077, *P* = 0.0062. Data are expressed as mean ± SEM (n = 5–7). *P < 0.05, **P < 0.01, ***P < 0.001

Recognizing synaptic plasticity as foundational for cognitive functions, we employed Golgi staining and electrophysiology recordings to decipher changes. No significant variations were found in total dendritic length, number of branching points, and intersections across groups (Fig. 6A, B and C, and 6E). However, the spine count plummeted in the IR + NS group **(**Fig. 6D F), an effect mitigated by liraglutide. Electrophysiology recordings further illustrated impaired LTP post-surgery, with liraglutide offering significant restoration **(**Fig. 6G H). These data show that liraglutide can effectively protect against the damage to synaptic plasticity caused by cardiac surgery.

## Discussion

Delirium is the most common neurological complication following cardiac surgery and is closely associated with an unfavorable prognosis (Patel et al. 2022). However, there are limited effective perioperative interventions to prevent or treat it. In our study, we discovered that cardiac surgery leads to mitochondrial dysfunction, NLRP3 inflammasome activation, and microglial activation. These changes subsequently cause an excessive engulfment of synapses and impairment of synaptic plasticity, culminating in delirium-like behaviors in aged mice. Notably, pretreatment with liraglutide significantly mitigates these alterations. This research further elucidates the mechanism of POD and offers a promising avenue for preventing POD using liraglutide.

A battery of behavioral tests was recently utilized to assess delirium-like behavior in a mouse model, facilitating preclinical delirium studies (Chen et al. 2022; Yang et al. 2022). We assessed mouse locomotor activity using the OFT and found significant impairment after cardiac surgery, in contrast to previous laparotomy-based studies (Li et al. 2021). This disparity may be attributed to the greater trauma and inflammatory response induced by cardiac surgery(Hovens et al. 2016). Furthermore, anxiety-like symptoms were evident 24 h after cardiac surgery, as detected by the EPM, aligning with earlier research findings (Cursano et al. 2021). To evaluate spatial and recognition memory, we employed the YMT and ORT. These assessments also gauge attention and high-level neurological function, given the fundamental role these factors play in memory formation. Notably, impairments in attention and memory, which are core symptoms of delirium, were observed in mice 24 h post-cardiac surgery, indicating significant delirium-like behaviors in aged mice following this procedure.

Clinical and preclinical evidence increasingly suggests that neuroinflammation is pivotal in the pathophysiology of delirium (Alam et al. 2018; Wilson et al. 2020). A hallmark of neuroinflammation is mitochondrial dysfunction, leading to the accumulation of ROS and consequent neural injury (Li et al. 2022; Song et al. 2021). Mitophagy, the selective removal of damaged mitochondria, acts as a protective mechanism, preserving the integrity of the mitochondrial pool and countering oxidative stress (Awasthi et al. 2021). The Pink1/Parkin pathway is central to mediating mitophagy(Lazarou et al. 2015). When mitochondria are damaged, they lose membrane potential, leading to Pink1 accumulation on the outer mitochondrial membrane. Subsequently, Pink1 recruits and activates the E3 ubiquitin ligase, Parkin, which ubiquitinates various proteins on the outer mitochondrial membrane. This process then interacts with LC3 and P62 to assemble the autophagosome, facilitating the degradation of the damaged mitochondria (Rüb et al. 2017). Established researches highlight that dysfunctional mitophagy significantly contributes to various neurodegenerative diseases, with enhanced mitophagy emerging as a promising prophylactic strategy (Kerr et al. 2017; Li et al. 2023; Lou et al. 2020). In our study, we observed that cardiac surgery triggered delirium-like behaviors in aged mice. This was paralleled by an upregulation in mitophagy. However, this upregulation appeared compensatory and inadequate. This assertion is supported by our findings that the administration of liraglutide further augmented mitophagy, ameliorating delirium-like behaviors.

The NLRP3 inflammasome is an intracellular multi-protein complex within innate immune cells that orchestrates the host’s inflammatory response, activated by a wide array of danger signals (Zhou et al. 2016). Upon activation, it recruits and activates Caspase-1, which in turn prompts the maturation and secretion of IL-1β(Okada et al. 2014). Prior research has indicated that dysregulation of mitophagy can trigger the activation of this complex, subsequently initiating microglia-mediated neuroinflammation (Lee et al. 2019; Zhou et al. 2011). In our study, we observed that cardiac surgery led to a pronounced activation of the NLRP3 inflammasome, concomitant with an upregulation of damaged mitochondria and activated microglia. Importantly, liraglutide treatment bolstered mitophagy, dampening the activation of both the NLRP3 inflammasome and microglia.

Dendritic spines are small protrusions emanating from neuronal dendrites, comprising the postsynaptic component of most excitatory synapses in the brain (Calabrese et al. 2006). PSD95 is the primary scaffolding protein within the excitatory postsynaptic density and plays a pivotal role in synaptic plasticity (Wu et al. 2017) Long-Term Potentiation (LTP), a specific form of synaptic plasticity, signifies an activity-driven augmentation in synaptic transmission between neurons, and is fundamental to learning and memory processes (Bin Ibrahim et al. 2022). A plethora of studies have shown that the over-activation of microglia can lead to the engulfment of synapses, culminating in synaptic loss and disruption of synaptic plasticity which is intimately tied to cognitive deterioration (Hong et al. 2016). Within the scope of our research, we discerned that cardiac surgery triggers pronounced microglial activation, prompting the pruning of excess synapses, resulting in synaptic loss and LTP impairment. The administration of liraglutide effectively counteracts this damage, hinting at its potential as an effective treatment in ameliorating POD.

In conclusion, our findings demonstrate that pretreatment with liraglutide mitigates delirium-like behaviors in aged mice subjected to cardiac surgery. This protective effect appears to be linked to the inhibition of NLRP3 inflammasome activation through the augmentation of mitophagy, subsequently reducing microglial activation and impairments in synaptic plasticity. This study not only sheds light on the pathophysiology underlying POD development but also offers a fresh clinical perspective on the potential of liraglutide in POD prevention.
