# Correction to: Liraglutide ameliorates delirium-like behaviors of aged mice undergoing cardiac surgery by mitigating microglia activation via promoting mitophagy

**DOI:** 10.1007/s00213-024-06538-4

**Published:** 2024-01-24

**Authors:** Min Jia, Xin Lv, Tong Zhu, Jin-Chun Shen, Wen-xue Liu, Jian-jun Yang

**Affiliations:** 1 Department of Anesthesiology, Jinling Hospital, Affiliated Hospital of Medical School, Nanjing University, Nanjing, China; 2 Department of Anesthesiology, Pain and Perioperative Medicine, The First Affiliated Hospital of Zhengzhou University, Zhengzhou, China; 3 Department of Thoracic and Cardiovascular Surgery, Institute of Cardiothoracic Vascular Disease, Affiliated Drum Tower Hospital of Nanjing University Medical School, Nanjing University, Nanjing, China

**Correction to** **:** **Psychopharmacology**

10.1007/s00213-023-06492-7

In the published paper, the authors would like to address inadvertent errors concerning the affiliations of the last author (Jian-jun Yang) and a footnote.

The corrected details are listed as follows:

The corrected information

Min Jia^1,2^, Xin Lv^1^, Tong Zhu^1^, Jin-Chun Shen^1^, Wen-Xue Liu^3^, Jian-Jun Yang^1,2^

1 Department of Anesthesiology, Jinling Hospital, Affiliated Hospital of Medical School, Nanjing University, Nanjing, China.

2 Department of Anesthesiology, Pain and Perioperative Medicine, The First Affiliated Hospital of Zhengzhou University, Zhengzhou, China.

3 Department of Thoracic and Cardiovascular Surgery, Institute of Cardiothoracic Vascular Disease, Affiliated Drum Tower Hospital of Nanjing University Medical School, Nanjing University, Nanjing, China.

In addition, the statement “Min Jia contributed equally to this work” was removed from the footnote.

The original article has been corrected.

